# An Integrated Artificial Cilia Based Microfluidic Device for Micropumping and Micromixing Applications

**DOI:** 10.3390/mi8090260

**Published:** 2017-08-24

**Authors:** Yu-An Wu, Bivas Panigrahi, Yueh-Hsun Lu, Chia-Yuan Chen

**Affiliations:** 1 Department of Mechanical Engineering, National Cheng Kung University, Tainan 701, Taiwan; n16041090@mail.ncku.edu.tw (Y.-A.W.); n18047068@mail.ncku.edu.tw (B.P.); 2 Department of Radiology, Taipei City Hospital, Zhongxing branch, Taipei 103, Taiwan; ataru.lu@gmail.com; 3 Department of Radiology, National Yang-Ming University, Taipei 112, Taiwan

**Keywords:** artificial cilia, micromixing, micropropulsion, micro-particle image velocimetry (µPIV), hydrodynamics

## Abstract

A multi-purpose microfluidic device that can be used for both micromixing and micropropulsion operations has always been in demand, as it would simplify the various process flows associated with the current micro-total analysis systems. In this aspect, we propose a biomimetic artificial cilia-based microfluidic device that can efficiently facilitate both mixing and propulsion sequentially at the micro-scale. A rectangular microfluidic device consists of four straight microchannels that were fabricated using the microfabrication technique. An array of artificial cilia was embedded within one of the channel’s confinement through the aforementioned technique. A series of image processing and micro-particle image velocimetry technologies were employed to elucidate the micromixing and micropropulsion phenomena. Experiment results demonstrate that, with this proposed microfluidic device, a maximum micromixing efficiency and flow rate of 0.84 and 0.089 µL/min, respectively, can be achieved. In addition to its primary application as a targeted drug delivery system, where a drug needs to be homogeneously mixed with its carrier prior to its administration into the target body, this microfluidic device can be used as a micro-total analysis system for the handling of other biological specimens.

## 1. Introduction

Microfluidics is the rapidly developing technology that deals with the control of fluid on micro-scaled platforms popularly known as microchannels. This technology is drawing substantial attention from academic researchers, as well as industrial groups, since it reduces the overall processing cost, time of experiments and usage of reagents compared to conventional laboratory techniques. Applications of this technology are immense, and range from chemical analysis [1] and disease detection [2,3] to soft robots [4]. To perform multiple functions (e.g., flow mixing, generation and detection) simultaneously, an integrated microfluidic system or device has been used that can be described as a network of microchannels such as micromixers, micropumps, and detection chambers [1,3]. For further miniaturization of these devices, it is indeed necessary to design and develop microchannels that can multitask.

Cilia are the greatest natural boon to living organisms; through them, organisms can manipulate, sense, and control their surrounding flow [5]. These extremely flexible and slender hair-liked organelles protrude from the outer membrane of mammalian cells, and usually have a length of between 2 and 15 µm. Functions of cilia are immense, and range from micro scale fluid manipulation and microorganism locomotion to cellular and developmental processes. According to their functionality, cilia can be broadly classified into two groups: motile (popularly known as moving cilia) and immotile cilia (alternatively known as primary cilia) [6]. Motile cilia use their flexible structure to propel the flow or facilitate particle motion along the cell’s surface. They are typically evidenced on the outer membrane of the paramecia, in the human respiratory tract, inside the fallopian tubes of females, etc. For instance, ciliary presence in the airways serves the purpose of sweeping dust particles and other pathogens back to the trachea that previously sticks to the mucus produced by the globule cells which allows the human to breathe easily without any exasperation. Non-motile cilia basically act as sensors, and are found in the ear, kidney, nervous system, etc. For instance, in the kidney, cilia sense the direction of urine flow and instruct the cells accordingly [7]. To generate fluid flow in an environment of low Reynolds number, motile cilia exhibit spatial, temporal, and even oriental asymmetry in their respective motions. For example, densely packed cilia on the surface of paramecia exhibit a synchronized back and forth motion; namely, recovery and effective strokes [5]. In the effective stroke, cilia beat in a straight path, while in the recovery stroke they roll back to their original position by moving close to the surface of the cells in a tangential manner. This nonreciprocal motion of ciliary beating results in spatial asymmetry, prompting fluid transportation. Moreover, cilia in the vertebrate embryonic nodes exhibit oriental asymmetry in their motion for fluid propulsion. As an example, cilia on the surface of mouse embryo nodes exhibit a tilted conical beat to transport the fluid around, determining the left-right symmetry that is crucial for vertebrate development [8,9].

Inspired by nature, artificial cilia have now been created in the laboratory to facilitate flow in microfluidic environments. These artificial cilia can be actuated under the influence of external stimuli—such as electric, magnetic, resonance or even light stimuli—and exhibit their capabilities for generating flow and mixing within the microfluidic platforms [10]. However, applications of the artificial cilia have certain limitations with respect to the electrical, optical or resonance fields because of their potential drawbacks. For example, with regard to a high electric field, electrolysis may occur, which can damage biological components such as red blood cells, and so on. In addition to this, Joule heating is one of the other major disadvantages associated with electrically actuated artificial cilia. Artificial cilia driven with optical and acoustic energy have a promising future. Still, relatively sophisticated components and manufacturing processes push them away from being an ideal candidate for real engineering practice. Magnetically actuated artificial cilia, on the other hand, can be considered as a good alternative, as they require a less complex fabrication process, in addition to their inert property towards biological specimens. Evans et al. 2007 presented a procedure for producing high-aspect ratio biomimetic cilia of PDMS-Ferro fluid composite material, and demonstrated that it can be actuated corresponding to a permanent magnetic field [11]. Since then, several artificial cilia-based microfluidic devices have been reported that can efficiently facilitate mixing [12,13], pumping [14,15,16,17,18,19] or even fluid flow sensing [20,21]. It was observed that with the optimized configuration, superior mixing can be achieved with these artificial cilia-based microfluidic devices in a very short time span, and with the consumption of a negligible amount of energy [12,13,22]. Numerically and experimentally, it was demonstrated that, with an optimized design, significant flow can be generated through these devices [16,17]. Hussong et al. 2011 experimentally investigated the flow induced through a rectangular-shaped, magnetically actuated artificial cilia-based device, and observed that the phase-averaged velocity reached a peak value of 130 µm/s. It was further highlighted that the reciprocal nature of artificial cilia motion results in an oscillatory flow [14]. Beating kinematics of magnetically actuated artificial cilia towards flow generation were investigated, and it was evidenced that correct positioning of defects along the filament length leads to significant improvement in flow generation [23]. In another experimental work, Shields et al. 2010 suggested that a maximum volumetric flow rate of 510 pL/s could be generated with these kinds of devices [24].

A multi-purpose microfluidic device that can facilitate both mixing and propulsion is always in demand, as it will simplify the process, cost, and chemical usages associated with the current practice in micro total analysis systems. Little research has been reported in this aspect. Kim et al. 2009 proposed a multifunctional device that uses an array of electrodes fabricated in a herringbone shape structure, demonstrating enhanced pumping and a better mixing simultaneously [25]. In another work, the researchers proposed a pneumatic microfluidic rotary device that is capable of fluid transportation and mixing [26]. Recently, Ding et al. 2014 numerically quantified the mixing and transport phenomena arising from an array of cilia beating in a metachronal pattern [27]. They observed fluid transportation on the cilia tip, and mixing in the region below the cilia tip. The obtained results were consistent with the results obtained in experiments conducted by another group of researchers, where they observed a similar phenomenon in artificial cilia-based microfluidic devices [24]. Moreover, these kinds of multi-purpose devices have a wide range of applications. Among them, one is targeted drug delivery systems, where it is essential to uniformly mix the drugs with their carrier prior to their administration into target system [28]. However, for such an application, uniform mixing and propulsion through the depth of the flow domain is necessary. We therefore propose an artificial cilia-based microfluidic device through which superior mixing and uniform propulsion can be achieved in a sequential manner. To elucidate the micromixing and propulsion phenomena quantitatively, a series of image processing and micro-particle image velocimetry (μPIV) methods were employed. In the end, applications of the present device are described in detail.

## 2. Materials and Methods

### 2.1. Design and Fabrication of Artificial Cilia-Based Microfluidic Device

**Figure 1:**
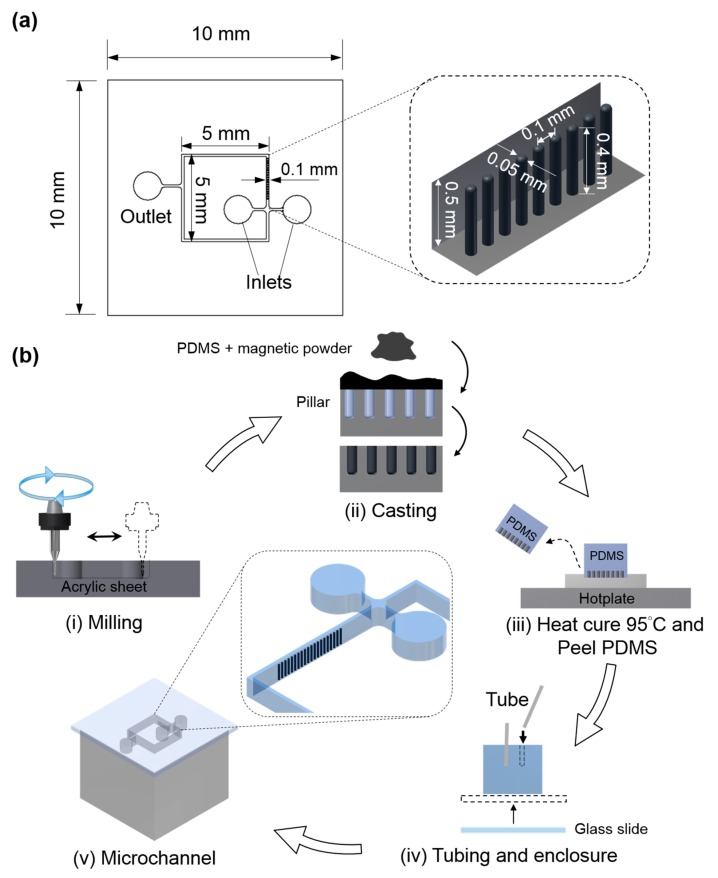
(**a**) Design and dimensional details of the microchannel (inset picture illustrates the dimensional details of artificial cilia); (**b**) Microfabrication flow process layout.

The design details of the multi-functional microfluidic device and artificial cilia are depicted in Figure 1a. As illustrated, the microfluidic device has two inlets for introducing different fluids for the micromixing operation. However, for the micropropulsion operation, both the inlets were designed to be closed in order to prohibit the access of fluid. This device consists of four straight channels, with an array of artificial cilia is embedded within one of those. The other channels are reserved for observing the dynamic responses of biological specimens susceptible to fluid rheology influences. The aspect ratio (height/diameter) of the artificial cilia was determined to be 8 to ensure its optimal functionality [12]. For the fabrication of the microfluidic device with artificial cilia embedded within, a series of computerized numerical control (CNC) micromachining processes, followed by PDMS casting, were employed (Figure 1b). The details are described in the following text. First, the geometric pattern of the microfluidic device was imprinted on an acrylic substrate (thickness of 5 mm) through a micro milling operation with an endmill 0.4 mm in diameter. Moreover, to drill the grooves for artificial cilia, a drill bit of 0.05 mm in diameter was used. To obtain the structure of artificial cilia, commercially available neodymium–iron–boron particles of 5 µm (MQP-15-7, Magnequench, Singapore) and Polydimethylsiloxane (PDMS, Sylgard 184, Dow Corning Corp., Midland, MI, USA) in a weight ratio of 1:4 were introduced into the grooves made for the artificial cilia. Hence, after degassing, PDMS were introduced into the mold prepared for the microfluidic channel. This was followed by a curing process through hot plate baking at a temperature of 95 °C. Approximately 4 hours later, the device was peeled from its parent mold. The array of artificial cilia was magnetized by manually contacting them with a permanent neodymium magnet. Once magnetized, a cover slip was added, enclosing the microchannel, and polyethylene tubes were attached to the respective inlets and outlets. Due to the induced residual stress during the microfabrication process, artificial cilia tips were found close to the inner side of the channel wall.

### 2.2. Magnetic Actuation System

**Figure 2:**
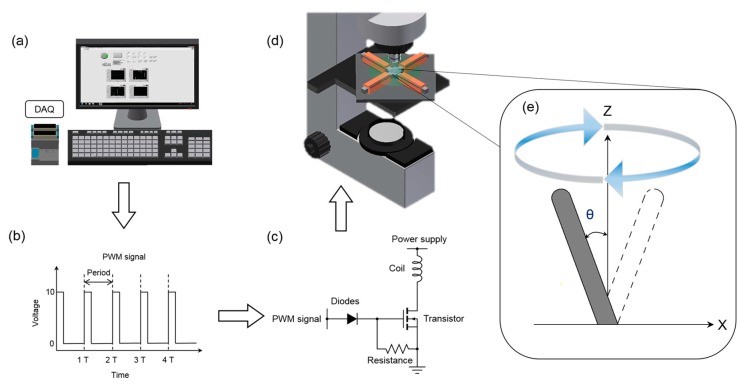
Schematic illustration of the magnetic actuation system. (**a**) Algorithm to generate the artificial cilia motion implemented through an in-house script; (**b**,**c**) Pulse-width modulation (PWM) signals were generated and applied via a switching circuit to manage the power supply; (**d**) Orientation of the microchannel corresponding to the actuation system and microscope (**e**) Schematic illustration of the artificial cilia trajectory.

To actuate artificial cilia in a continuous cyclic manner, a magnetic actuation system was employed (Figure 2). This actuation system consists of a custom-made graphical user interface, four magnetic coil sets, data acquisition system (NI cDAQ-9174, National Instruments, Austin, TX, USA) with input modules, switching circuit and a power supply (GPR-3510HD DC Power Supply, Instek, Taiwan). Each individual magnetic coil was built by wrapping approximately 1200 turns of single-strand 24 gauge enameled magnetic wire onto a hexagonal iron bar 90 mm in length, which further served as an electromagnet corresponding to an external power supply. To achieve the desired circular beating patterns of the artificial cilia, sine and cosine functions were applied along *x* and *y*-directions through an in-house script via the graphical user interface. A modulated pulse-width modulation (PWM) signal was implemented through a switching circuit to control the time duration and the amplitude of power supply. It should be noted that the proposed actuation system can generate a magnetic field up to 0.2 T. A detailed description of the actuation system can be found elsewhere [29].

### 2.3. Flow Visualization

To perform the flow mixing operation, two glycerol aqueous dyed and colorless (with 75% by weight) solutions were introduced to the microchannel through a syringe pump (KD Scientific, Inc., Holliston, MA, USA) at a constant flow rate of 0.8 µL/min. A series of image processing techniques were adopted and implemented through the imaging process software Image J [30]. In post-image processing, the mixing performance was evaluated by quantifying the intensity difference downstream of the microchannel. The details about the algorithm to find mixing performance can be found elsewhere [19].

To quantify the generated flow propulsion through the microfluidic device, micro-particle image velocimetry (μPIV) analysis was used. Fluorescent polystyrene particles (Microgenics, Inc., Fremont, CA, USA) 3.2 μm in diameter were seeded into deionized water and introduced in the microfluidic device. For the instantaneous flow visualization, a fluorescent microscope (BX60, Olympus Corp., Tokio, Japan) was incorporated. To quantify the instantaneous velocity, commercially available PIV software (Dynamic Studio, Dantec Dynamics, Skovlunde, Denmark) was used. An adaptive PIV with 16 × 16-pixel interrogation window was employed to quantify velocity vectors. It should be noted that both the inlets and outlet were sealed during the propulsion experiment to ensure flow stabilization. To attune propulsion, artificial cilia were actuated, and the flow generated due to artificial cilia motion was recorded in the observation zone.

## 3. Results and Discussions

**Figure 3:**
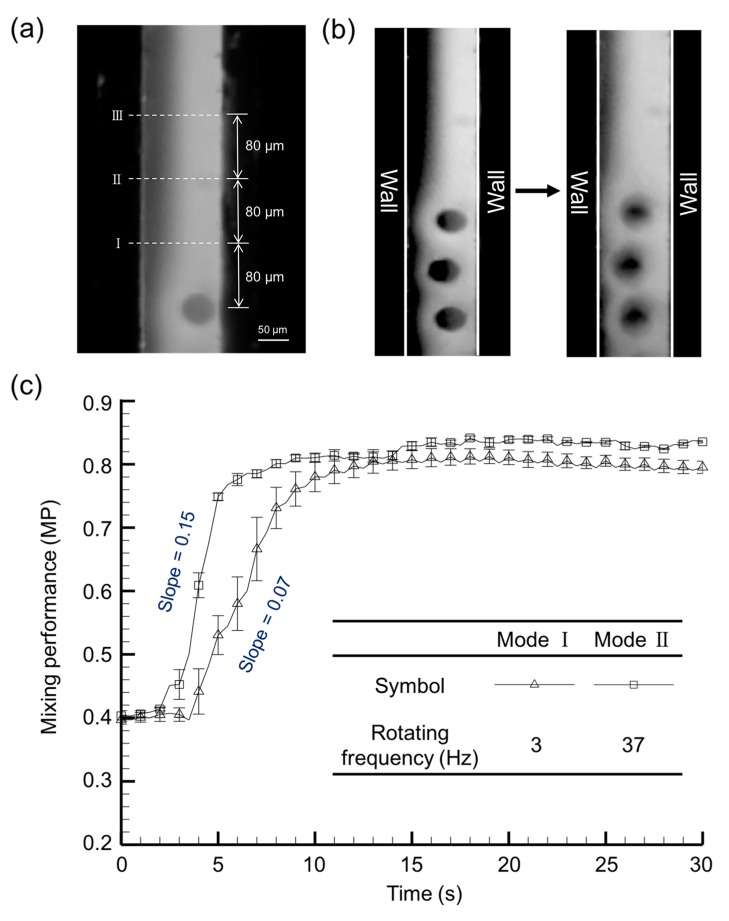
(**a**) Three zones, situated at distances of 80, 160 and 240 µm from the last cilia position, respectively, were selected to quantify the mixing performance; (**b**) Pictures of the flow field when the cilia are static and rotating at frequency of 37 Hz; (**c**) Time dependent micro-mixing performance (MP) in two distinctive artificial cilia beating modes (e.g., 3 and 37 Hz).

The mixing performance of the microfluidic device at two artificial cilia beating frequency values (3 and 37 Hz) was quantified, and is illustrated in Figure 3. To avoid terminological confusion, they were coined Mode I and Mode II, respectively, and are referred to throughout the manuscript accordingly. It can be observed that the scanning area (area covered by the path traversed by the tip of the artificial cilia) for the artificial cilia at the two modes have similar magnitudes of 7468 µm^2^ and 6532 µm^2^, respectively. In order to quantify the mixing performance of the microfluidic device, three different zones of area 32 × 45 µm^2^ were selected, located at distances of 80, 160, and 240 µm from the terminally located artificial cilia at the plane, respectively, where the stream of two fluids interact with each other. Measurement depth was set at 100 µm below the microchannel top, which is consistent with the height of artificial cilia. To quantify the mixing performance, grey-level deviation of the recorded images of each zone were calculated [19]. The final value of the mixing performance was quantified between 0 and 1, where 0 denotes no mixing, while 1 denotes complete mixing. It was observed that, when the artificial cilia were static (left column of the Figure 3b), a boundary was exhibited near the intersection of the two types of fluids. In contrast, once the artificial cilia started rotating, this layer disappeared and the two fluid streams were mixed (see right column of the Figure 3b). The mixing performances of the proposed microfluidic device during the two distinct modes are illustrated in Figure 3c. Corresponding to the highly viscous medium, a mixing performance (MP) of 0.4 was evidenced at the start of experiment (<5 s), when the artificial cilia were in a static position. This low initial MP explains the importance of the micromixing operation for highly viscous fluids in environments with a low Reynolds number (*Re* = 1.16 × 10^−3^). It was evidenced that the mixing performance upsurged to a value of 0.75 from its initial value of 0.4 after the actuation of artificial cilia (i.e., in a time period between 5 to 10 s). In this transient region, a larger magnitude of slope (mixing performance/time) and smaller magnitude of root-mean-square deviation (RMSE) values were observed for Mode II. This further indicates that, compared to Mode I, complete and homogeneous mixing was achieved with Mode II in a relatively shorter period time. In other words, Mode II is 1.6 times faster than the Mode I, in terms of mixing speed. For both modes, uniform mixing was achieved after 10 s. Maximum mixing performance of mode II and mode I was found to be 0.84 and 0.81, respectively. It can be summarized that, at the higher artificial rotational frequency, uniform and stable mixing can be achieved in a very competitive time span.

**Figure 4:**
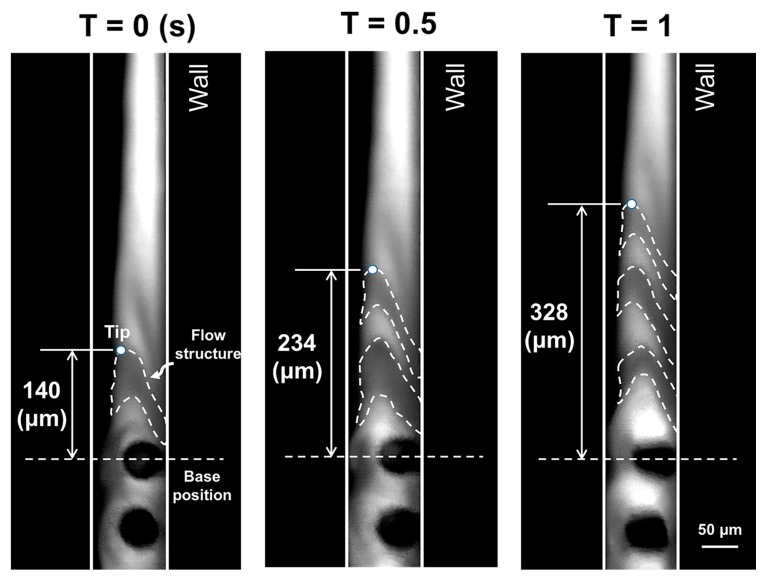
Time lase imaging of the flow profile illustrates the migration of the flow structure at the plane of cilia tip.

Aside from the micromixing task, the proposed device was tested for its application in micropropulsion. Time lapse imaging of the flow profile near the tip of the artificial cilia during the rotational operation reveals the migration of eccentric flow structures in this wall-bounded flow (Figure 4). The real-time recording is provided in the Supplementary Materials. To quantify the migration of these flow structures, the tip of the first flow structure was marked and traced corresponding to a base position marked near to the terminally located artificial cilia. It can be observed that these flow structures traversed a distance of 188 µm in a period of 1 s, which illustrates an obvious propagation of the flow field with the rotational beating behaviour of artificial cilia. To understand the underlying flow propulsion phenomena in a more quantitative manner, the next section is devoted to the study of the flow field hydrodynamics during propulsion.

**Figure 5:**
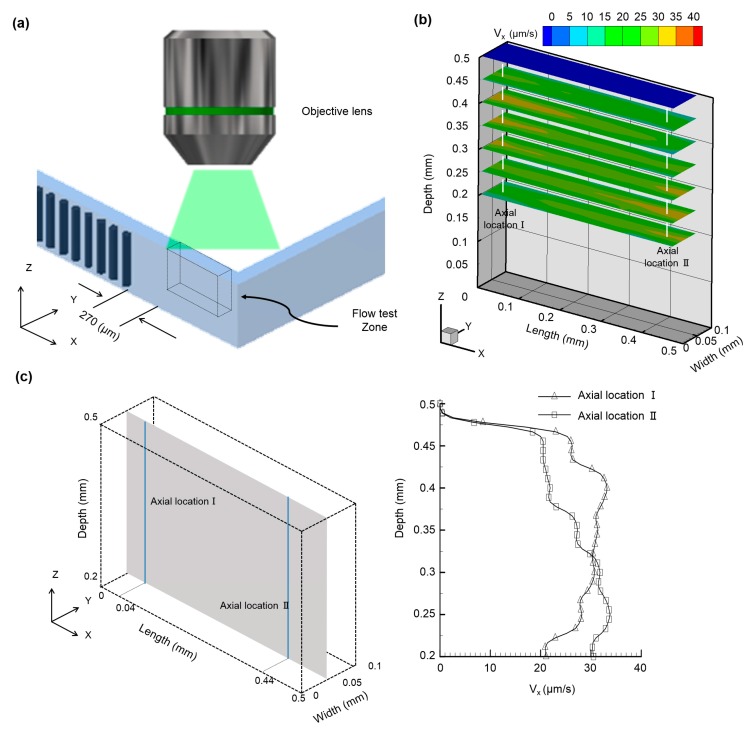
(**a**) Schematic illustration of the microfluidic device depicting the observation zone, which is situated at a distance of 270 µm from the terminally located artificial cilia; (**b**) Calculated ensemble streamwise velocity distribution of the six selected horizontal planes along the depth of the microfluidic device, situated at distances of 200 µm, 250 µm, 300 µm, 350 µm, 400 µm, 450 µm, 500 µm, respectively, from the bottom of the microfluidic device. Two different velocity profiles were evidenced at two axial locations situated at distance of 310 µm (Axial location I) and 710 µm (Axial location II) away from the rearmost artificial cilia; (**c**) Quantified instantaneous velocity profiles at the two different axial locations (i.e., axial location I and axial location II) of the observation zone along the vertical mid plane of the channel width.

To investigate the propulsion induced due to the beating of the artificial cilia array, µPIV analysis was carried out. An observation zone with an area of 500 × 100 µm^2^, located at a distance of 270 µm from the terminal artificial cilia, was selected (Figure 5a). To achieve superior micropropulsion, the magnitude of the artificial cilia rotating frequencies were pre-meditated to 40 Hz; the details of the beating trajectory and its working mechanism can be found elsewhere [15,29]. The flow test zone was selected away from the artificial cilia array based on the hypothesis that flows in this zone are stable and free from any kind of oscillation induced by the continuous beating of the artificial cilia array. In-plane induced flow patterns of the six horizontal planes in the measuring zones along the depth of the microfluidic device were selected for local flow field visualization. The ensemble streamwise velocity distribution is depicted for all six horizontal planes (Figure 5b). Two types of flow phenomenon were observed in the six selected horizontal planes, and in order to quantify them, two axial locations, close to and distant from the last artificial cilia along the depth of the microfluidic device, were selected from the six horizontal planes and denoted as axial location I and axial location II. It was observed that, for axial location I, a velocity of greater magnitude was observed near to the cilia tip, and that the magnitude of velocity decreased along with the depth of the artificial cilia. These results were consistent with the findings of other researchers, in which similar flow phenomena were observed near the artificial cilia tip [24]. In contrast to this, at axial location II—that is, away from the array of artificial cilia—a distinct flow phenomenon was observed, where maximum velocity was observed on the lower horizontal plane, which was 250 µm away from the bottom of the microchannel. To delineate the flow phenomena in detail, the in-depth velocity profiles at both axial locations are quantified and illustrated in Figure 5c. As observed, the absolute peak velocities at axial location I and axial location II were found to be 33.2 µm/s and 33.8 µm/s at different horizontal planes of 400 µm and 250 µm, respectively. At this point, it can be deduced that the flow at axial location I was partially affected by the actuation of the artificial cilia. Meanwhile, as the fluid traverses and reaches axial location II, the no-slip boundary condition contributes more significantly to the flow, leading to a more parabolic velocity profile with a velocity peak at 250 µm in depth.

**Figure 6:**
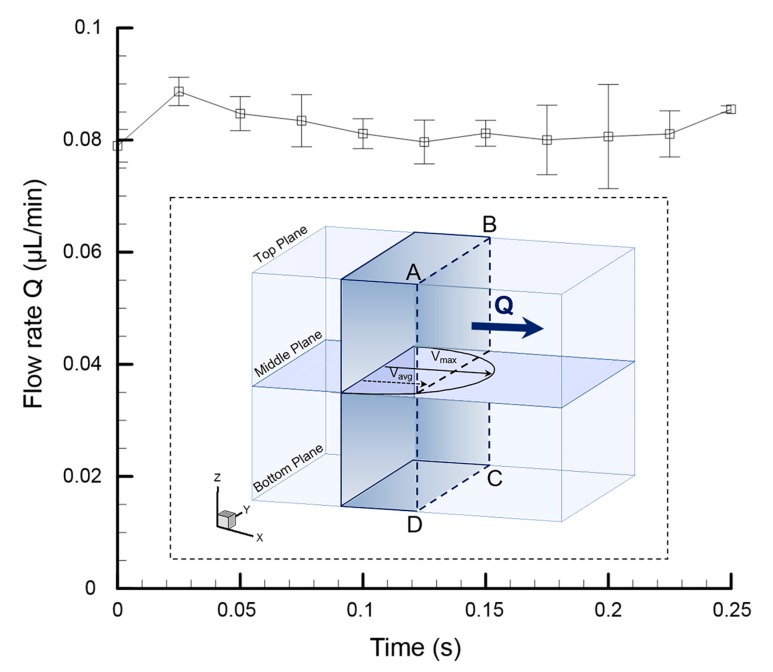
The net flow generated through the microfluidic device with respect to time illustrates a stable flow generated through the device. The inset picture describes the flow rate quantified by multiplying the instantaneous average flow velocity with the cross-sectional area of the microchannel.

As mentioned in the previous paragraph, in contrast to the previous findings, where propulsion was only observed near the cilia tip [24,27], in this experiment, a fully developed parabolic velocity profile was obtained in the region away from where the artificial cilia are located. To quantify the net flow rate (*Q*) generated through the proposed device, instantaneous average velocity (*V*_avg_) at the middle of the plane was calculated and multiplied by the cross-sectional area of the microfluidic device (Figure 6(ABCD)). This statement holds true, assuming the flow inside the microfluidic device is steady, with no wall slip condition. To find out the instantaneous net velocity generated by the artificial cilia array, it is essential to eliminate the back flow velocity generated due to the reciprocal nature of the ciliary motion. More details regarding the methodology for this can be found in one of our recently published articles [18]. A maximum flow rate of 0.089 µL/min was achieved through this proposed microfluidic device, which is competitive with the performance achieved by its counterparts [15,24]. For instance, in terms of fluid transport, through our proposed device, an average amount of 0.082 µL can be propagated per minute, which is approximately three times more than the amount of fluid transported with other artificial cilia-based microfluidic devices [24].

## 4. Conclusions

In this work, we demonstrated an artificial cilia-based microfluidic device that is capable of facilitating both micromixing and micropropulsion operations sequentially. A superior micromixing and stable propulsion capacity were evidenced through the proposed microfluidic device at the same budget cost, with minimal energy spent. In addition to that, in the cilia region, propulsion was evidenced in the tip region, which is in accordance with the findings described in the literature. However, away from the ciliary region, an in-depth parabolic flow profile was evidenced. This device will be particularly beneficial for the further design of targeted drug delivery systems, and work on this aspect is currently being carried out.

## Supplementary Materials

The following are available online at www.mdpi.com/2072-666X/8/9/260/s1, Figure S1: The array of artificial cilia situated closer to one microchannel wall due to the residual stress induced on them during the microfabrication procedure. Corresponding to the channel width of 100 µm the average artificial cilia distance from the closest wall found to be 40.03 ± 8.79 µm. The position of the artificial cilia has a crucial role to play towards the micropropulsion by inducing asymmetry on the flow field due to the influence of the wall while the cilia beats near to it; Figure S2: (a) Velocity contour along the x-direction on the top plane depicts the maximum velocity at the center of the plane. (b) Comparison between theoretical data and µPIV analysis data at a plane near the top of the microchannel illustrates a high degree of relevance. As illustrated both the data exhibit a high correlation of *R*^2^ = 0.99; Video S1: Video for Micropopulsion.
