# Supplementary material for: An Integrated Artificial Cilia Based Microfluidic Device for Micropumping and Micromixing Applications
Source: Micromachines (Basel). 2017 Aug 24;8(9):260. doi: 10.3390/mi8090260 (PMC6190408; doi:10.3390/mi8090260)
Supplement: Supplementary file 1 [file micromachines-08-00260-s001.zip › micromachines-189242 suppl final.pdf]

Supplementary Materials: An Integrated Artificial Cilia Based Microfluidic Device for Micropumping and Micromixing Applications

Yu-An Wu, Bivas Panigrahi, Yueh-Hsun Lu and Chia-Yuan Chen

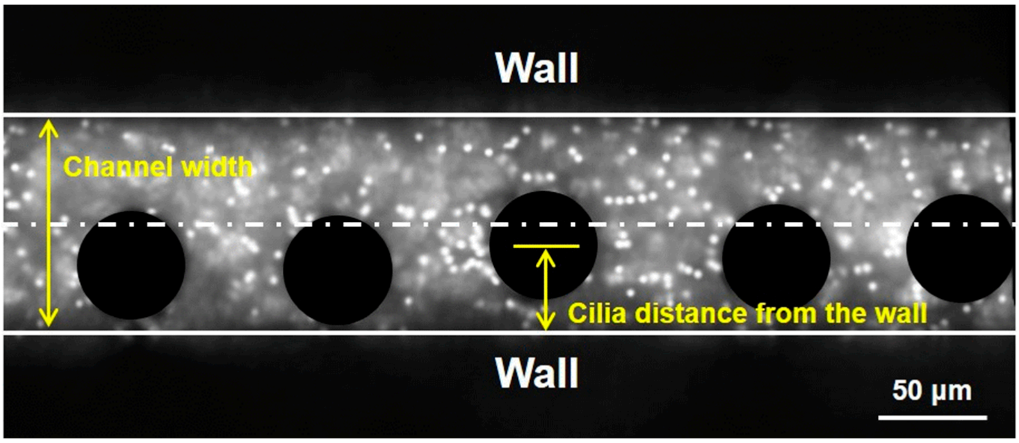

Figure S1. The array of artificial cilia situated closer to one microchannel wall due to the residual stress induced on them during the microfabrication procedure. Corresponding to the channel width of 100 μm the average artificial cilia distance from the closest wall found to be 40.03 ± 8.79 μm. The position of the artificial cilia has a crucial role to play towards the micropropulsion by inducing asymmetry on the flow field due to the influence of the wall while the cilia beats near to it.

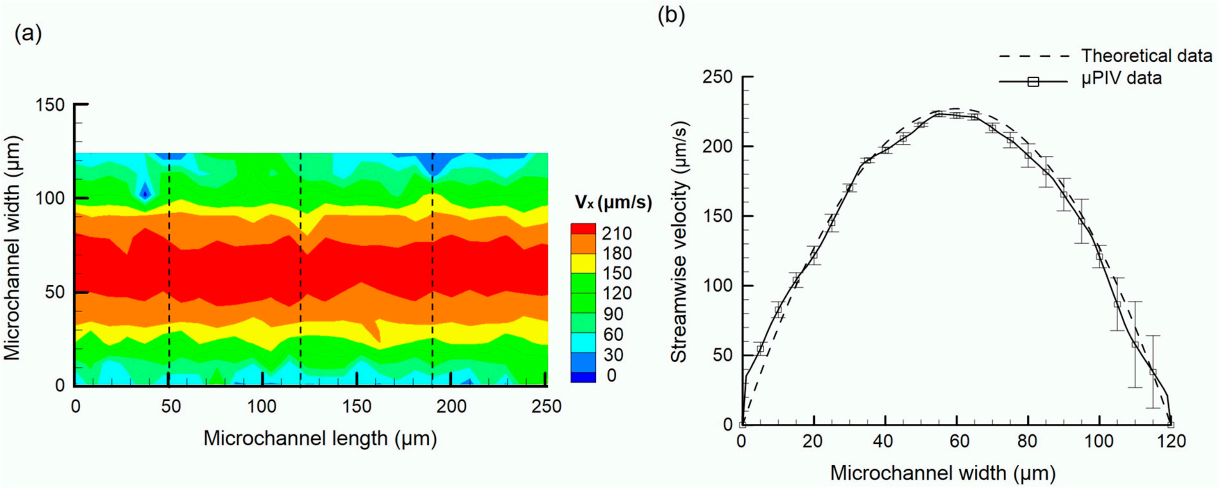

Figure S2. (**a**) Velocity contour along the X-direction on the top plane depicts the maximum velocity at the center of the plane. (**b**) Comparison between theoretical data and μPIV analysis data at a plane near the top of the microchannel illustrates a high degree of relevance. As illustrated both the data exhibit a high correlation of $R^2 = 0.99$.
